# Proteasome Accessory Factor C (*pafC*) Is a novel gene Involved in *Mycobacterium* Intrinsic Resistance to broad-spectrum antibiotics - Fluoroquinolones

**DOI:** 10.1038/srep11910

**Published:** 2015-07-03

**Authors:** Qiming Li, Longxiang Xie, Quanxin Long, Jinxiao Mao, Hui Li, Mingliang Zhou, Jianping Xie

**Affiliations:** 1 Institute of Modern Biopharmaceuticals, State Key Laboratory Breeding Base of Eco-Environment and Bio-Resource of the Three Gorges Area, Key Laboratory of Eco-environments in Three Gorges Reservoir Region, Ministry of Education, School of Life Sciences, Southwest University, Beibei, Chongqing 400715, China; 2 The Second Affiliated Hospital and the Key Laboratory of Molecular Biology of Infectious Diseases of the Ministry of Education, Chongqing Medical University, 1 Medical Road, Yuzhong District, Chongqing, 400016, China

## Abstract

Antibiotics resistance poses catastrophic threat to global public health. Novel insights into the underlying mechanisms of action will inspire better measures to control drug resistance. Fluoroquinolones are potent and widely prescribed broad-spectrum antibiotics. Bacterial protein degradation pathways represent novel druggable target for the development of new classes of antibiotics. Mycobacteria proteasome accessory factor C (*pafC*), a component of bacterial proteasome, is involved in fluoroquinolones resistance. *PafC* deletion mutants are hypersensitive to fluoroquinolones, including moxifloxacin, norfloxacin, ofloxacin, ciprofloxacin, but not to other antibiotics such as isoniazid, rifampicin, spectinomycin, chloramphenicol, capreomycin. This phenotype can be restored by complementation. The *pafC* mutant is hypersensitive to H_2_O_2_ exposure. The iron chelator (bipyridyl) and a hydroxyl radical scavenger (thiourea) can abolish the difference. The finding that *pafC* is a novel intrinsic selective resistance gene provided new evidence for the bacterial protein degradation pathway as druggable target for the development of new class of antibiotics.

Tuberculosis, caused by *Mycobacterium tuberculosis*, remains a leading cause of mortality and morbidity worldwide^1^. One-third of the global population is latently infected with *M. tuberculosis* and millions die annually due to active tuberculosis^2^. Multidrug-resistant tuberculosis (MDR-TB) and extensively drug-resistant tuberculosis (XDR-TB) have worsened this scenario^3^,^4^. *M. tuberculosis* is intrinsically tolerant to most antibiotics largely due to the imperviousness of its unusual mycolic acid-containing cell wall^5^ to most chemotherapeutics^6^, and a wealth of efflux pumps^7^. Some genes involved in metabolism also can mediate intrinsic resistance in mycobacterium. The inactivation of asparagine synthetase AsnB, an asparagine biosynthetic enzyme catalyzing the transfer of the γ-amino residue of glutamine to the carboxyl residue of aspartate, dramatically sensitized *M. smegmatis* to multiple antibiotics, including rifampin, erythromycin, novobiocin, and fusidic acid^8^. It is imperative to find novel therapeutic targets or new effective antibiotics against tuberculosis.

Fluoroquinolones are important second-line drugs for the treatment of tuberculosis, and the new generation of fluoroquinolones (moxifloxacin) is becoming an important antituberculosis agent as both a first-line therapeutic^9^ and a second-line treatment for multidrug-resistant tuberculosis^10^,^11^. With the widespread administration of quinolones, the incidence of fluoroquinolone-resistant *M. tuberculosis* kept rising^12^. *M. tuberculosis* DNA gyrase, encoded by gyrA and gyrB, is well estabilished quinolone target^13^. Mutations within the highly conserved region, the so-called quinolone resistance-determining region (QRDR) of *gyrA*/*gyrB*, have been reported to be responsible for ≥70% of FQ resistance in clinical *M. tuberculosis* isolates^14^.

Intracellular protein degradation is essential for almost all organisms. For most organisms, there are two canonical intracellular degradation ways targeting unwanted proteins, namely proteolysis in lysosome and an ubiquitin-dependent process. While in *M. tuberculosis*, a prokaryotic ubiquitin-like protein (Pup) can tag the unwanted proteins, which is catalyzed by proteasomal accessory factor A (*pafA*) to form an isopeptide bonds between the γ carboxyl group of Pup glutamate and the side chain amine of lysine of protein substrate^15^. The Pup-proteasome system (pps) is essential for the virulence and persistence of pathogen within host. The widespread of PPS-system among non-pathogens implicates additional roles in cellular processes, which is reinforced by the supportive role of proteasome-mediated amino acid recycling for the survival of mycobacteria under nutrient limitation^16^. The essentiality of the *M. tuberculosis* proteasome pathway enabled it ideal drug target^17^. *pafA* is cotranscribed with two genes, namely *pafB* and *pafC*, which are dispensable for the function of *M. tuberculosis* proteasome^15^,^18^. However, the detailed function of *pafC* remains elusive.

In this study, we characterized the function of *pafC*. Using the *M. smegmatis pafC* mutant we isolated, we found that *pafC* might play a role in the intrinsic resistance to fluoroquinolones. The *pafC* deficiency potentiated the lethality of fluoroquinolones, such as moxifloxacin, norfloxacin, ofloxacin, ciprofloxacin, and environmental stress, such as hydrogen peroxide. Both an iron chelator (bipyridyl) and a hydroxyl radical scavenger (thiourea) can lower the moxifloxacin lethality, while moxifloxacin at low concentration did not have such effect. In brief, we firstly characterized the Proteasome Accessory Factor C (*pafC*), and found its potential role in the intrinsic resistance of mycobacterium to fluoroquinolones. Reactive oxygen species were proposed to mediate the function of *pafC*.

## Results

### Isolation and characterization of hypersensitive mutant M371 for fluoroquinolones

**Figure 1:**
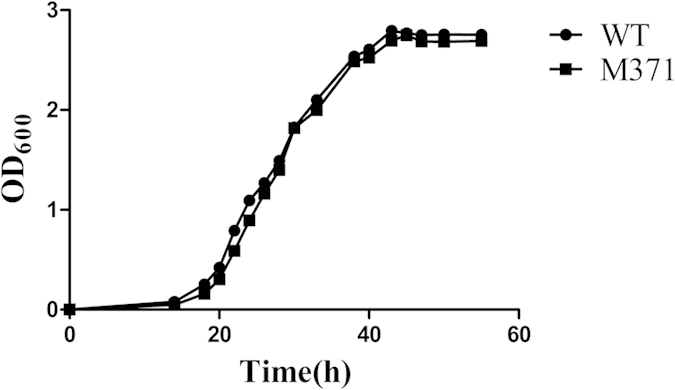
Growth of *M. smegmatis* mc^2^155 and M371. *Mycobacterium smegmatis* mc^2^155 and M371 were grown in Middlebrook 7H9 medium supplemented with 0.05% Tween80 and 0.2% glycerinum. The OD_600_ were determined at an interval of 4 h.

**Figure 2:**
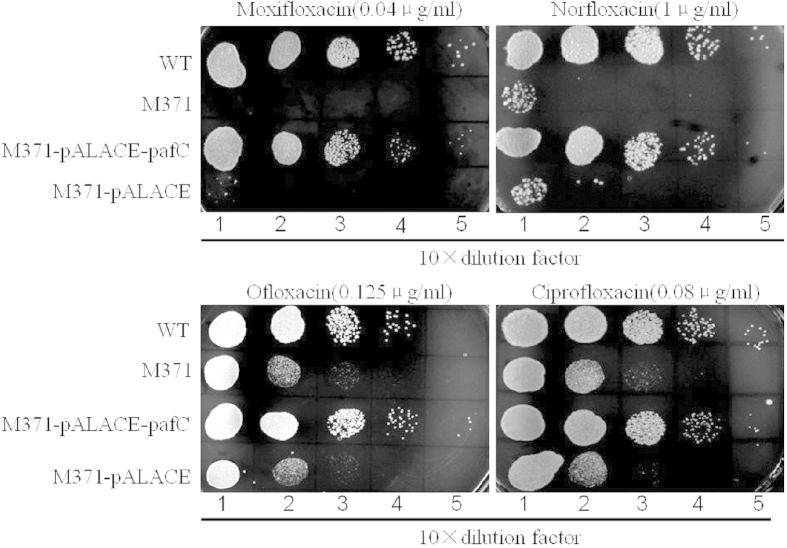
Growth of *M. smegmatis* mc^2^155 and M371 under fluoroquinolones exposure. Ten-fold serial dilutions of wild-type, M371, M371-pALACE-*pafC* and M371-pALACE were spotted on Middlebrook 7H10 containing indicated concentration of moxifloxacin, norfloxacin, ofloxacin and ciprofloxacin. Then the result was recorded when incubated at 37 °C for 3 days.

To identify mycobacteria genes involved in the intrinsic resistance to fluoroquinolones, we screened fluoroquinolones sensitive isolates using sublethal concentrations of moxifloxacin and obtained one mutant (named M371) hypersensitive to moxifloxacin from the constructed transposon insertion mutant library of *M. smegmatis* mc^2^155. No evident growth defect can be spotted when mutant cell was grown in 7H9 medium supplemented with 0.05% Tween80 and 0.2% glycerinum (Fig. 1). The mutant of M371 exhibited increased sensitivity to grow on 7H10 plate supplemented with indicated concentration of moxifloxacin, norfloxacin, ofloxacin and ciprofloxacin (Fig. 2), but its sensitivity to other antibiotics, including isoniazid, rifampicin, spectinomycin, chloramphenicol, capreomycin, remained the same (Fig. S1). To confirm the hypersensitivity of the mutant, MICs of M371 were determined in 7H9 liquid medium at 37 °C supplemented with antimicrobials at indicated concentrations with 2–4 fold increment (Table 1). No MIC difference between wild type *M. smegmatis* (WT) and M371 was found for ciprofloxacin. One possible interpretation is their difference is minor and less than 2-folds (Table 1). The results confirmed that M371 is hypersensitive to fluoroquinolones. Fatty acids, the major components critical for cell wall permeability, did not show significant difference between the WT and M371 by GC-MS analysis (Fig. S2).

### Mutant M371 is an *M. smegmatis* mc^2^155 *MSMEG_3888* disruptant

**Figure 3:**
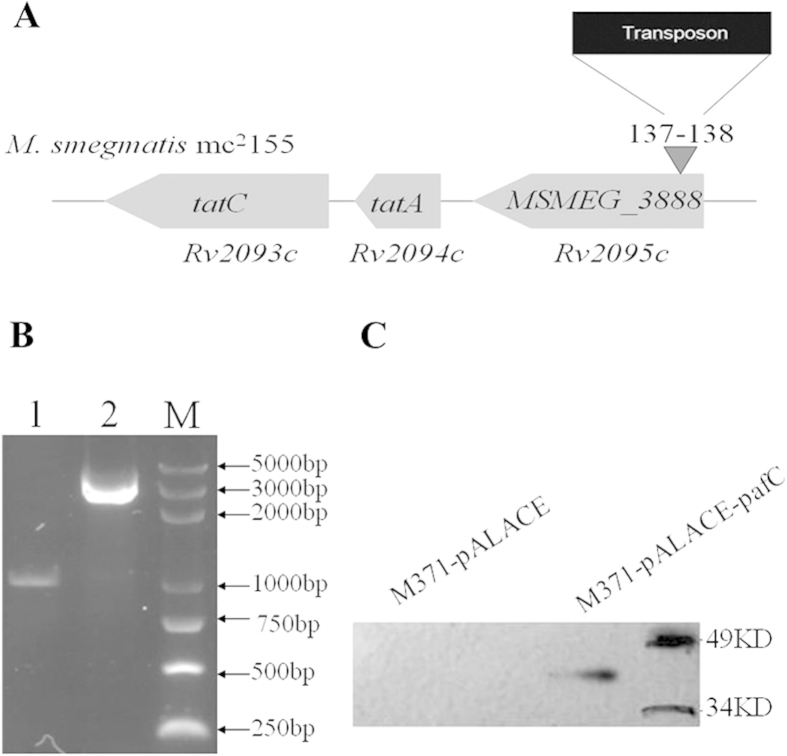
Genomic locus of the ΦMycoMar insertion and the construction of complement strain. (**A**) The *MSMEG_3888* gene and flanking genes are depicted. Gray arrows represent open reading frames and the black arrow indicates insertion of the transposon to generate the hypersensitive muant M371. (**B**) PCR amplification of *MSMEG_3888* using genomic DNA from *M. smegmatis* mc^2^155 (lane 1) and M371(lane 2). (**C**) Lysates were prepared from complement strain and subjected to Western blot to detect His-tagged PafC protein using mouse anti-His antibody, the lysate of M371-pALACE as a control.

To determine the molecular mechanism underlying the intrinsic fluoroquinolones resistance of mycobacteria in M371, plasmid rescue was used to locate and identify the disrupted gene. Results showed that transposon inserted a CA dinucleotide, 137 bp downstream from the ATG start codon of an open reading frame (ORF) of *MSMEG_3888*, which is predicted to encode a 318-amino acid polypeptide (Fig. 3A). To confirm this, we PCR amplified this gene using *M. smegmatis* mc^2^155 and M371 genome as template respectively. Results showed that an additional 2 kb product present in the M371 genome instead of *M. smegmatis* mc^2^155 (Fig. 3B). Taken together, the M371 mutant is an *MSMEG_3888* transposon insertion. BLAST results showed *MSMEG_3888* homologs are ubiquitous among actinomycetes, including *M. tuberculosis* (69% amino acid identity, *pafC*), *M. marinum* M (68%), *Nocardia brasiliensis* (56%) (Fig. S3). *Paf*C forms an operon with *pafB* and *pafA* in *Mycobacterium* and *Corynebacterium*. However, this operon was intervened by two genes in *Streptomyces* and *Nocardia* (Fig. S4). There is a conserved WYL domain in PafC via query in Conserved Domain Datebase (CDD) of NCBI (Fig. S5).

### Complementation of mutant M371

To establish the causality between the hypersensitive phenotype of M371 and mutation of *MSMEG_3888*, instead of secondary mutation or polar effects on downstream genes, we cloned the *M. tuberculosis paf*C gene into a shuttle vector pALACE^19^. The recombinant plasmid pALACE-*pafC* and empty vector pALACE were transformed into M371 by electroporating to prior obtained recombinant strains. Western Blot analysis using the anti-His antibody further confirmed the presence of the expressed ~40 kDa PafC-His fusion protein in the cell lysates of the complemented strain, and absence in the parental strain (Fig. 3C). The recombinant strains M371-pALACE-*pafC* and M371-pALACE were spotted on Middlebrook 7H10 containing various fluoroquinolones. The results showed that the *paf*C gene of *M. tuberculosis* complemented the mutant phenotypes of M371, but M371-pALACE failed to grow (Fig. 2). These results suggested that *pafC* is the gene underlying the fluoroquinolones intrinsic resistance.

### A pafC deficiency can potentiate the fluoroquinolones lethality

**Figure 4:**
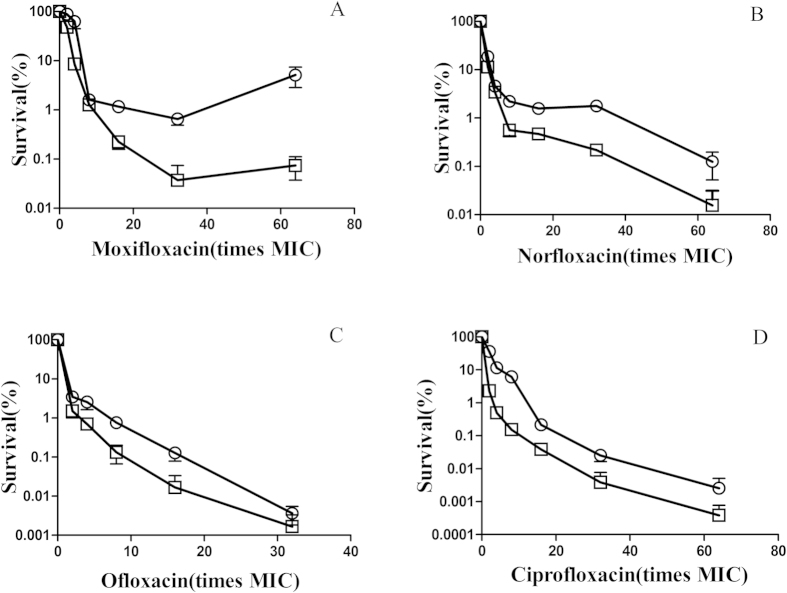
The effects of *pafC* deficiency on bacterial survival after antimicrobials treatment. Wild-type strain (*M. smegmatis* mc^2^155, OD_600_ = 1) and its *pafC* mutant strain (M371, OD_600_ = 1) were diluted (1:100) in 7H9 medium and then treated with the indicated concentrations of moxifloxacin for 2 h (panel **A**), the indicated concentrations of norfloxacin for 4 h (panel **B**), the indicated concentrations of ofloxacin for 4 h (panel **C**), the indicated concentrations of ciprofloxacin for 4 h (panel **D**). Symbols: open circles, wild type; open squares, *pafC* mutant. Percent survival was determined as in Methods. Error bars indicate standard deviation; similar results were obtained in replicate experiments.

**Figure 5:**
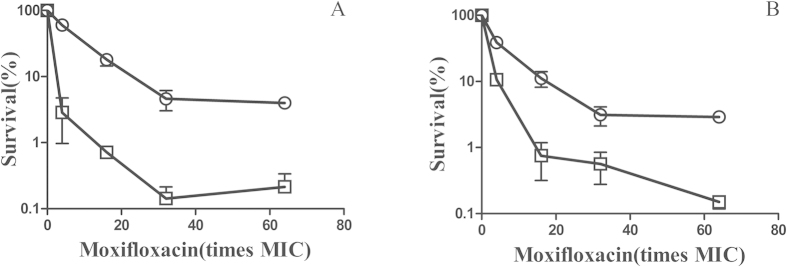
Mid-exponential phase culture (WT and M371, OD_600_ = 1) were diluted in 7H9 medium and then treated with the indicated concentrations of moxifloxacin for 2 h. (**A**) diluted with 1:25, (**B**) diluted with 1:10. Symbols: open circles, wild type; open squares, *pafC* mutant. Error bars indicate standard deviation; similar results were obtained in replicate experiments.

The gene of *pafC* encode a proteasome accessory factor C in mycobacterium, forming an operon with *Rv2096c* (*pafB*) and *Rv2097c* (*pafA*)^18^. *pafA* encoded pup ligase is responsible for pup conjugation to substrates subject to intracellular protein degradation^15^. PafA is essential for *M. tuberculosis* survival under RNI exposure *in vitro* and virulence *in vivo*^20^. *PafB* and *pafC* was speculated to play a role in RNI resistance^18^. To determine whether *pafC* deficiency will compromise the survival during lethal fluoroquinolones stress, we exposed *M. smegmatis* and M371 cells to fluoroquinolones. When wild-type and *pafC*-deficient cells were treated with various concentration of moxifloxacin for 2 h (Fig. 4A), survival rate of the *pafC* mutant was 10 to 20-fold lower than that of wild-type cells. Similar phenomenon was observed when wild-type and *pafC* mutant were treated with norfloxacin, ofloxacin and ciprofloxacin for 4 h (Fig. 4B–D). The *pafC* mutation can potentiate the lethal action of fluoroquinolones. Similar survival rates were observed between WT and M371 treated with moxifloxacin even when the concentration of bacteria was increased (Fig. 5). Both rifampicin and isoniazide are first-line anti-TB drugs, the survival of WT and M371 exposure to both drugs was compared. Slight difference was observed when treated with rifampicin, but not with isoniazide (Fig. S6).

### pafC is involved in the rapid killing effect of H_2_O_2_

**Figure 6:**
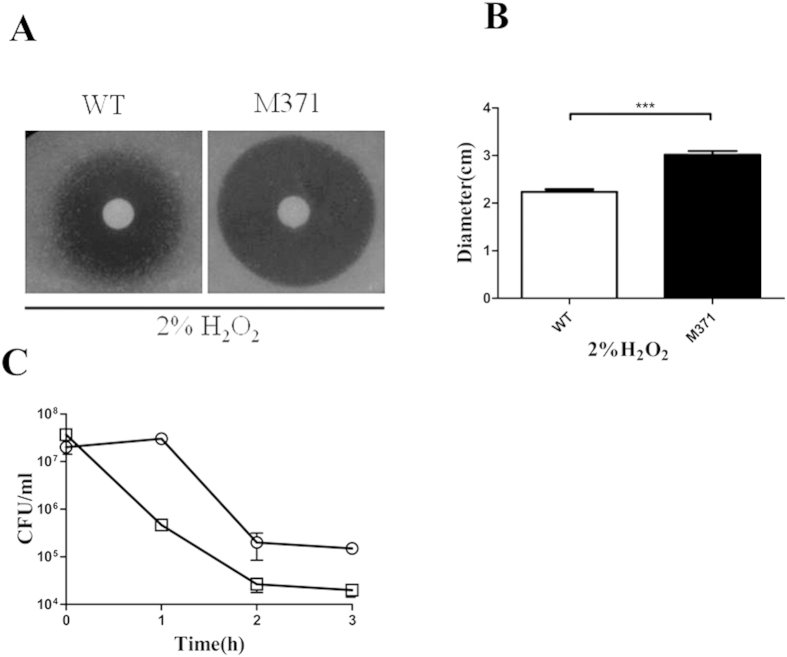
The effect of pafC deficiency on bacterial survival after treatment with H_2_O_2_. (**A**) Mid-exponential-phase culture were prepared as in Methods, 10 μl of 2% H_2_O_2_ were spotted on the Whatman disk. (**B**) After overnight incubation, the diameter of zone of complete inhibition was measured. (**C**) Wild-type strain (*M. smegmatis* mc^2^155, open circle) and its *pafC* mutant strain (M371, open squares ) were treated with the indicated times for 5 mM H_2_O_2_. Symbols: open circles, wild type; open squares, *pafC* mutant. Error bars indicate standard deviation; similar results were obtained in replicate experiments.

A previous study has shown that fluoroquinolones stimulate the production of highly deleterious hydroxyl radicals in Gram-negative and Gram-positive bacteria, which ultimately contribute to cell death^21^. H_2_O_2_ is an important molecule highly reactive and can react with iron to generate hydroxyl radicals from Fenton reaction^22^. To determine whether the sensitivity of M371 to fluoroquinolones is caused by reactive oxygen species, we examined the effect of *pafC* deficiency on the lethality of oxidative stress. The survival rates were compared between wild-type and *pafC* deficient cells using disk diffusion antibiotic sensitivity testing (Fig. 6A). The results showed that M371 was more sensitive than wild-type against stress (Fig. 6B). To further confirm *pafC* mutant was responsible for the H_2_O_2_ sensitivity, wild-type and M371 were exposed to 5 mM H_2_O_2_ for various incubation duration. The results showed that M371 survival declined by 10–100 fold, while the wild-type cells remained viable till 1 h after exposure (Fig. 6C). Taken together, these results unequivocally demonstrated that *pafC* deficiency increases the lethal effect of H_2_O_2_.

### The addition of thiourea or 2, 2’-bipyridyl can abrogate or attenuate the lethality of moxifloxacin

**Figure 7:**
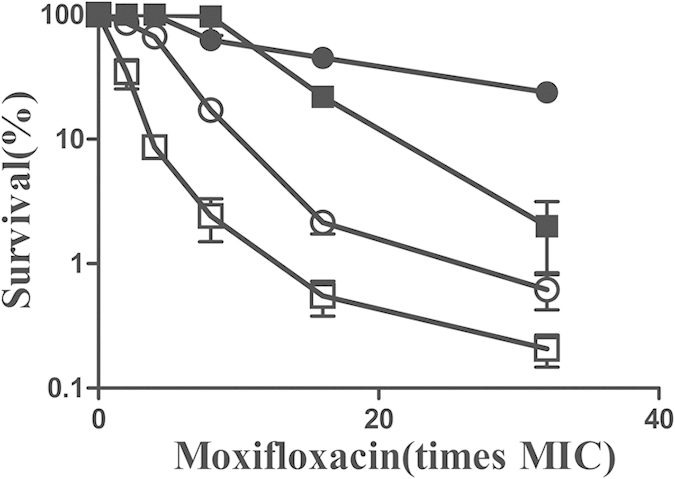
The effects of thiourea and 2, 2’-bipyridyl on moxifloxacin lethality. Exponentially growing *M. smegmatis* cells were preincubated with 0.25 mM bipyridyl and 100 mM thiourea for 10 min before they were treated with various concentration of moxifloxacin for 2 h. Symbols: open circles, wild type; open squares, *pafC* mutant; filled circles, wild type plus bipyridyl and thiourea; filled squares, *pafC* mutant plus bipyridyl and thiourea. At least three replicate experiments were performed, and all had results similar to those shown.

To further test that hydroxyl radicals contribute to the lethality of moxifloxacin for M371 and wild-type, exponentially growing cells were treated with 2, 2'-bipyridyl (an iron chelator) that interferes with the Fenton reaction and with thiourea, a hydroxyl radical scavenger. Both thiourea and bipyridyl can protect the M371 strain and wild-type cells from moxifloxacin. The same lethality rate was observed between M371 and wild-type at low concentration of moxifloxacin when treated with thiourea or bipyridyl, but less protective to M371 than to wild-type cells at high concentration (Fig. 7). The observed incomplete protection might be due to of the application of subinhibitory concentration of thiourea and bipyridyl that were unable to completely eliminate hydroxyl radical accumulation. Thus, these results suggested that the deficiency of *pafC* can potentiate the lethality of several fluoroquinolones and might be the result of accumulated hydroxyl radicals.

## Discussion

Emerging drug resistant tuberculosis represents formidable challenge to global public health. Intensive studies have unveiled a multitude of mechanisms of action underlying such drug resistance in addition to drug-specific targets, such as efflux pumps^23^ of MFS family^24^,^25^,^26^,^27^,^28^, ABC family^29^,^30^,^31^ and RND Family^32^. These efflux proteins can mediate broad-spectrum resistance to streptomycin, rifampicin, fluoroquinolones, ethambutol, ethionamide, isoniazid and other compounds. Metabolic and signaling related genes are recently reported to be involved in intrinsic resistance to multiple antituberculosis drugs, such as asparagine synthetase asnB, protein kinase G, isocitrate lyase, a key enzyme of glyoxylate shunt^8^,^33^,^34^,^35^. These findings enabled more druggable targets for future antibiotics development.

In this study, we identified mycobacteria proteasome accessory factor C (*pafC*) as a novel gene underlying intrinsic resistance to fluoroquinolones. Fluoroquinolones are important antibiotics for tuberculosis treatment. Wild-type mycobacteria are intrinsic resistant to fluoroquinolones and drug efflux pump^30^,^36^,^37^ or pentapeptide protein MfpA^38^ are reported to be responsible for this resistance. We demonstrated that inactivation of the *pafC* gene in *M. smegmatis* confers hypersensitivity to fluoroquinolones including moxifloxacin, norfloxacin, ofloxacin and ciprofloxacin, and *M. tuberculosis* counterpart can restore the phenotype, indicating that mycobacteria *pafC* is involved in the intrinsic resistance to fluoroquinolones. To the best of our knowledge, this is the first time that *pafC* has been reported to be involved in fluoroquinolones intrinsic resistance.

We also found that the effect of *pafC* on the fluoroquinolones sensitivity is related to reactive oxygen species. Two pathways are proposed to interpret the bacteriocidal effect of the new generation of fluoroquinolones. One is chloramphenicol-sensitive pathway and depend on hydroxyl radical, the other is chloramphenicol-insensitive lethal pathway^39^. The *pafC* mutant is more sensitive to H_2_O_2_ than wild type *M. smegmatis*. The protective assay of thiourea and bipyridyl further confirmed that the accumulation of reactive oxygen species played a role in *pafC* mediated fluoroquinolones sensitivity. The different sensibility of M371 between various fluoroquinolones (Fig. 2) might be associated with the structure or ability to produce reactive oxygen species. The production of reactive oxygen species (ROS) is proposed to be a common mechanism of cellular death induced by bactericidal antibiotics^21^. Though the idea that antibiotics kill bacterial cells through the action of ROS was challenged by others^40^,^41^, it has been supported by a number of follow-up studies^42^,^43^,^44^,^45^. Recent studies show that lethal attacks from bacterial and viral species also result in ROS production in target cells^46^. The slight survival rate difference between WT and M371 observed when treated with rifampicin might be due to the formation of hydroxyl radical induced by rifampicin in *M. tuberculosis*^47^. However, the mechanism underlying the role of hydroxyl radical in *pafC* mutant hypersensitive to fluoroquinolones remains to be further illuminated. Three genes, *pafA*, *pafB* and *pafC*, were previously identified to be in an operon and all three genes appear to play a role in RNI resistance^18^. *M. tuberculosis* is continually exposed to endogenous reactive oxygen species (ROS) as part of normal aerobic respiration, as well as exogenous ROS and reactive nitrogen species (RNS) generated by the host immune system in response to infection^48^. Our finding may provide further evidence that *pafC* enhances mycobacterial survival within macrophages.

The biochemical characteristics of *pafC* remain elusive. The only anecdotal clue for its biochemical function is the conserved WYL domain in *pafC*. WYL domain bearing transcription factors are reported to regulate the expression of the defense systems unless a specific ligand is present either to derepress or activate transcription^49^. Further biochemical characterization of *pafC* will help elucidate the role of *pafC* in intrinsic drug resistance. To our knowledge, Compartmentalized proteases are often involved in regulating the stability of transcription factors, directly or indirectly^50^,^51^. Notably a recent study showed that defects in proteasome-dependent degradation resulted in transcriptional changes in *M. tuberculosis*^52^. Thus, we speculate that the phenomenon we observed as a result of *pafC* mutant may affect transcription factors and metal homeostasis.

The intrinsic drug resistance of Mycobacterium, in particular the *M. tuberculosis*, represents formidable obstacle to tuberculosis treatment. The finding that *pafC* mediating intrinsic resitance to the important fluoroquinolones implicates that novel compounds inhibiting the *pafC* function might represent ideal potentiators of fluoroquinolones. The limited and conserved distribution of *pafC* in actinomycetes suggests that such new inhibitors might be narrow spectrum and will not disturb the normal flora.

## Methods

### Bacterial strains, plasmid, and growth conditions

*M. smegmatis* mc^2^155 and M371 were grown in Middlebrook 7H9 medium supplemented with 0.05% Tween80 and 0.2% glycerinum or Middlebrook 7H10 plates supplemented with 0.5% glycerinum. Luria-Bertani medium was used to culture *E. coli* strains. Antibiotic were added at following concentrations: ampicillin, 100 μg/ml; kanamycin, 50 μg/ml for *E. coli* and 20 μg/ml for *M. smegmatis* mc^2^155; hygromycin, 75 μg/ml for *E. coli* or 50 μg/ml for *M. smegmatis* mc^2^155. All cultures were incubated at 37 °C.

### Construction and screen of *M. smegmatis* mc^2^155 ΦMycoMar insertion library

The transposon system ΦMycoMarT7 was utilized to construct a transposon insertion mutant library of *M. smegmatis* mc^2^155 as described previously^53^,^54^. For phage infection, *M. smegmatis* mc^2^155 cells were grown to late-log phase in Middlebrook 7H9 broth without antibiotics. Cells were pelleted, washed twice with mycobacteriophage buffer (50 mM Tris-HCl pH 7.5, 150 mM NaCl, 10 mM MgSO_4_, 2 mM CaCl_2_), and then resuspended in the same buffer. Phage was added at a multiplicity of infection of 10:1, and the cells and phage were incubated at 37 °C for 4 h to allow infection to occur. The bacteria were then plated on Middlebrook 7H10 agar supplemented with kanamycin (20 μg/ml) and incubated at 37 °C for 3-4 days. Kanamycin-resistant (i.e., transposon-containing) *M. smegmatis* colonies were patched onto Middlebrook 7H10 agar to obtain a library of 3500 clones. To screen this library, clones were replica plated onto Middlebrook 7H10 agar supplemented with moxifloxacin at one-third the MIC for the WT *M. smegmatis* mc^2^155. Clones that failed to grow on the drug-containing plates were deemed hypersensitive. The MIC was determined to further confirm the drug hypersensitive of obtained clone.

### Localization of the ΦMycoMar insertion

Plasmid rescue was used to localize and identify the disrupted gene as previously described^53^,^55^. Total chromosomal DNA of transposon insertion mutant was digested with *Sac*II. Digested DNA was self-ligated with T4 DNA ligase and transformed into competent *E. coli* DH5α cells. Plasmid DNA was isolated from Km^r^ *E. coli* DH5α cells. The primer of 5′-GCCTTCTTGACGAGTTCTTCTGAG-3′ was used to determine the DNA sequence of the MycoMar/chromosomal junction. These DNA sequences were compared with the *M. smegmatis* mc^2^155 genome. The primers of flanking sequence were designed to further determine the location of transponson.

### Cloning the gene of *pafC* from *M. tuberculosis*

The gene of *pafC* from *M. tuberculosis* H37Rv was amplified by PCR using the forward primer 5′-GAGCCATATGATGAGCGCCCTGT-3′ and reverse primer 5′-TTAATCGATTCACGGCGGCGCAGCT-3′. The target gene was inserted into the *Nde*I and *Cla*I of pALACE which contains a hygromycin resistance cassette as described^19^. The recombinant plasmid was sequenced to confirm the right clone and named pALACE-*pafC* . Plasmids pALACE-*pafC* and pALACE were transformed into mycobacterial cells by electroporation according to standard protocol. Transformants were selected on Middlebrook 7H10 agar containing hygromycin (50 μg/ml).

### Antimicrobial susceptibility assays

Four methods were used for measuring antibiotic or H_2_O_2_ effect.

#### Spot tests

Wild type *M. smegmatis* mc^2^155 and mutant strains were grown to an A_600_ of 0.8–1.0 tested for their susceptibility to antibiotics by spotting a 10-fold serial dilution initially on Middlebrook 7H10 (Difco) plates containing a range of drug: moxifloxacin (0.04 μg/ml), norfloxacin (1 μg/ml), ofloxacin (0.125 μg/ml), ciprofloxacin (0.08 μg/ml), isoniazid (8 μg/ml), rifampicin (4 μg/ml), spectinomycin (32 μg/ml), chloramphenicol (16 μg/ml), capreomycin (2 μg/ml).

#### MIC

Growth inhibition (MIC) was determined by broth dilution with visual inspection of a series of tubes each containing about 10^5^ bacteria in 1 ml of 7H9 medium supplemented with concentrations of drug increasing by 2 times increments. Following 3 days incubation at 37 °C, the lowest concentration that prevented visible growth was defined as the MIC.

#### Disk diffusion method

The disk diffusion method was used to qualitatively measure the differences in H_2_O_2_ sensitivities between wild type and mutant mycobacterium. Mid-exponential-phase cultures were used to prepare the lawns of cells as previously described^56^. An indicated amount of H_2_O_2_ was spotted on 5.5 mm-diameter Whatman filter disks placed on the bacterial lawn. After overnight incubation, the diameter of zone of complete inhibition was measured. All the experiment was repeated at least three times.

#### Survival curves

Mid-exponential phase culture were diluted in 7H9 medium and grown at 37 °C treated for various times and at various concentrations with antibiotics, and surviving cells were estimated by colony formation on drug-free agar. The percentage cfu recovered was determined relative to an untreated control sampled at the time when antibiotics added. The effect of hydroxyl radicals on fluoroquinolones-mediated lethality was assessed by treating cells with subinhibitory concentration of 2,2'-bipyridyl and thiourea were added to bacterial cultures, followed by fluoroquinolones treatment for indicated times and at indicated concentrations. All the CFU data are listed in the supplementary materials.

## Additional Information

**How to cite this article**: Li, Q. *et al*. Proteasome Accessory Factor C (*pafC*) Is a novel gene Involved in *Mycobacterium* Intrinsic Resistance to broad-spectrum antibiotics - Fluoroquinolones. *Sci. Rep*. **5**, 11910; doi: 10.1038/srep11910 (2015).

## Supplementary Material

Supplementary Information

Supplementary Information

## Figures and Tables

**Table 1:** MIC of various antibiotics for *M. smegmatis* mc^2^155 and M371.

Strains	MIC (μg/ml)				
	Moxifloxacin	Norfloxacin	Ofloxacin	Ciprofloxacin	Nalidixic acid
*M. smegmatis* mc^2^155	0.05	4	0.25	0.125	128
M371	0.025	1	0.125	0.125	32
