# Supplementary material for: Proteasome Accessory Factor C (pafC) Is a novel gene Involved in Mycobacterium Intrinsic Resistance to broad-spectrum antibiotics - Fluoroquinolones
Source: Sci Rep. 2015 Jul 3;5:11910. doi: 10.1038/srep11910 (PMC4490553; doi:10.1038/srep11910)
Supplement: Supplementary Information [file srep11910-s1.pdf]

Proteasome Accessory Factor C (*pafC*) Is a novel gene Involved in *Mycobacterium* Intrinsic Resistance to broad-spectrum antibiotics – Fluoroquinolones

Qiming Li[1,3], Longxiang Xie[1,3], Quanxin Long[1,2], Jinxiao Mao[1], Hui Li[1], Mingliang Zhou[1] and Jianping Xie[1*]

[1]Institute of Modern Biopharmaceuticals, State Key Laboratory Breeding Base of Eco-Environment and Bio-Resource of the Three Gorges Area, Key Laboratory of Eco-environments in Three Gorges Reservoir Region, Ministry of Education, School of Life Sciences, Southwest University, Beibei, Chongqing 400715, China

[2]The Second Affiliated Hospital and the Key Laboratory of Molecular Biology of Infectious Diseases of the Minis try of Education, Chongqing Medical University, 1 Medical Road, Yuzhong District, Chongqing, 400016, China

[3]These authors contributed equally to this work.

*Corresponding author: Jianping Xie: georgex@swu.edu.cn

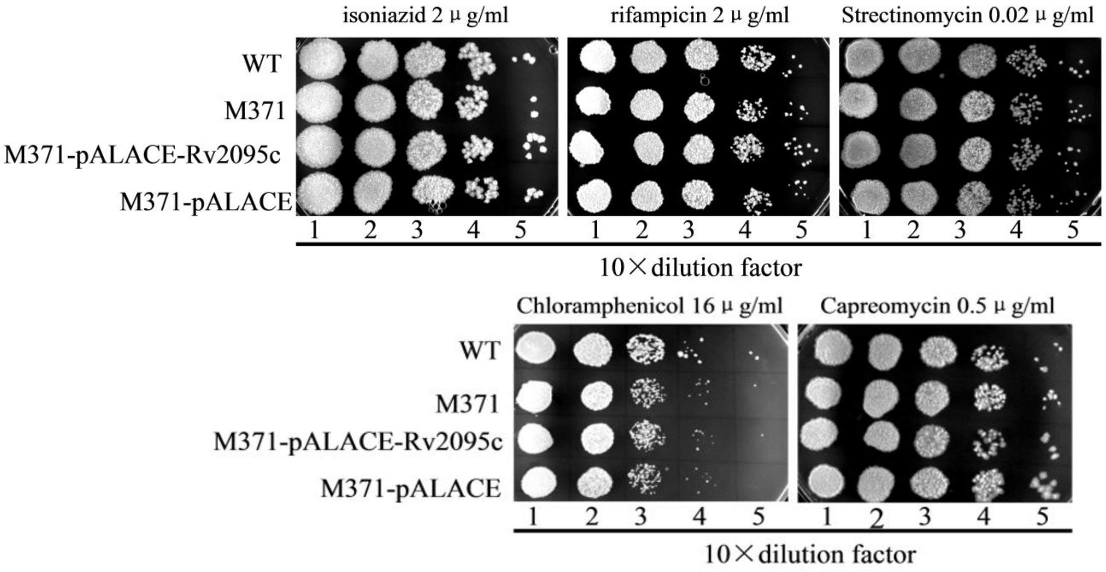

Supplementary Figure. 1. Growth of *M. smegmatis* mc^{2}155 and M371 under antibiotics exposure.

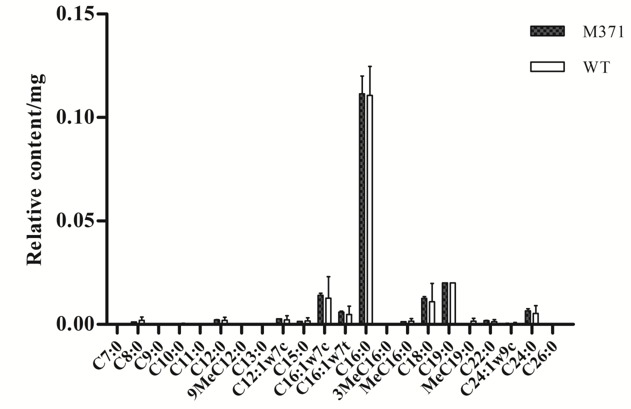

Supplementary Figure. 2. GC-MS analysis of aliphatic acid in M371 and WT.

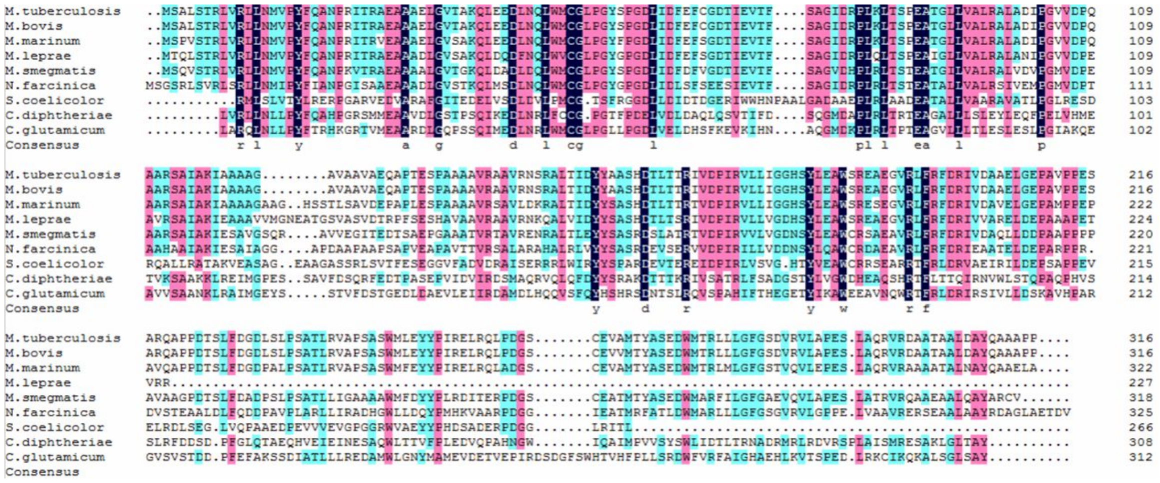

Supplementary Figure. 3. Amino acid sequence alignment (generated using DNAMAN) of pafC in mycobacteria and close relatives. Completely conserved residues are shaded in blue.

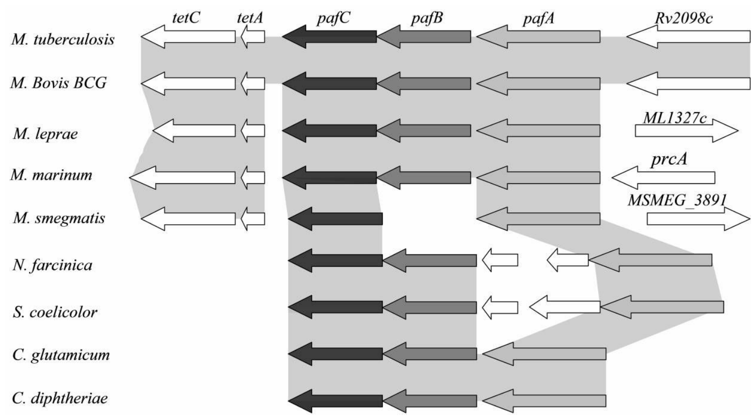

Supplementary Figure. 4. Genomic context of *pafC* in mycobacteria and close relatives. The grey shading represents regions of conservation between genomes. Arrows represented with a dark grey background correspond to genes that are conserved among all species, whereas genes without ortholog in at least one species are shown with white arrows.

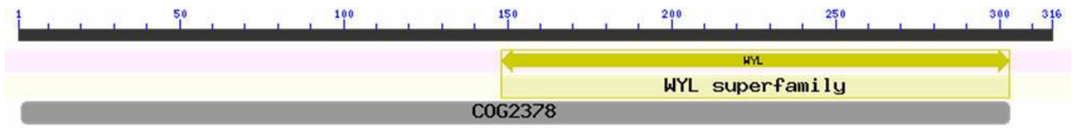

Supplementary Figure. 5. Conserved domain analysis of *pafC* from *M. tuberculosis* in the Conwerved Domain Database.

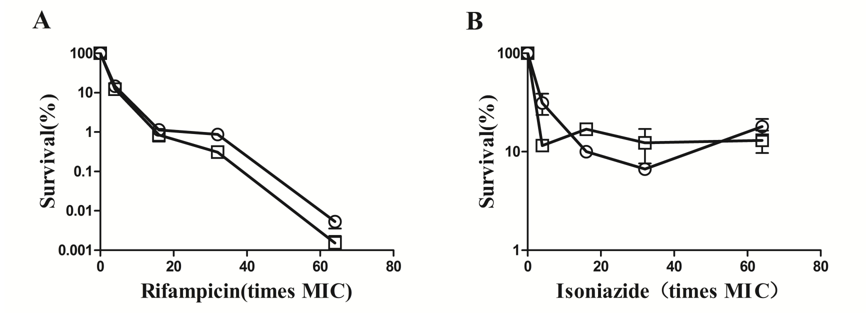

Supplementary Figure. 6. Wild-type strain (*M. smegmatis* mc^{2}155, OD_{600}=1) and its pafC mutant strain (M371, OD_{600}=1) were diluted (1:100) in 7H9 medium and then treated with the indicated concentrations of rifampicin (panel A) and isoniazide (panel B) for 12h.
